# An Orbital Angular Momentum (OAM) Mode Reconfigurable Antenna for Channel Capacity Improvement and Digital Data Encoding

**DOI:** 10.1038/s41598-017-10364-4

**Published:** 2017-08-29

**Authors:** Baiyang Liu, Guoying Lin, Yuehui Cui, RongLin Li

**Affiliations:** South China University of Technology, School of Electronic and Information Engineering, Guangzhou, 510640 China

## Abstract

For purpose of utilizing orbital angular momentum (OAM) mode diversity, multiple OAM beams should be generated preferably by a single antenna. In this paper, an OAM mode reconfigurable antenna is proposed. Different from the existed OAM antennas with multiple ports for multiple OAM modes transmitting, the proposed antenna with only a single port, but it can be used to transmit mode 1 or mode −1 OAM beams arbitrary by controlling the PIN diodes on the feeding network through a programmable microcontroller which control by a remote controller. Simulation and measurement results such as return loss, near-field and far-field radiation patterns of two operating states for mode 1 and mode −1, and OAM mode orthogonality are given. The proposed antenna can serve as a candidate for utilizing OAM diversity, namely phase diversity to increase channel capacity at 2.4 GHz. Moreover, an OAM-mode based encoding method is experimentally carried out by the proposed OAM mode reconfigurable antenna, the digital data are encoded and decoded by different OAM modes. At the transmitter, the proposed OAM mode reconfigurable antenna is used to encode the digital data, data symbol 0 and 1 are mapped to OAM mode 1 and mode −1, respectively. At the receiver, the data symbols are decoded by phase gradient method.

## Introduction

It is well known that the electromagnetic waves can carry both linear and angular momentum. Angular momentum (AM) can be divided into spin angular momentum (SAM) and orbital angular momentum (OAM). The OAM is related to the beam vorticity and phase singularity, which can provide unlimited orthogonal modes and offer an additional degree of freedom in communication systems^1^. In 1992, Allen *et al*. found that light beams with the transverse azimuthal dependence of exp(−*jlφ*) carry orbital angular momentum which has unbounded states and can be used to increase the spectrum efficiency^2^, but only recently the OAM beams are used to improve channel capacity in the near-field zone line-of-sight (LOS) link at radio frequency. In 2014, a high-capacity millimeter-wave communication system with orbital angular momentum multiplexing and polarization multiplexing is proposed^3^. However, there are some criticisms about OAM are raised, the usefulness of OAM is reduced. On one hand, researchers classifying OAM-based communication systems as a special case of MIMO systems^4^. On the other hand, OAM multiplexing does not work in the far field, high-order OAM modes decay as 1/*r* ^l^ where l is the order of OAM mode. Nevertheless, OAM waves can remarkably increase the channel capacity in the near-fields zone without any signal processing. Moreover, some researchers propose to use different OAM modes to encode and decode the digital data^5^.

Multi-OAM-mode beams generated by a single aperture antenna has attracted more and more attentions in recent years, because of its ability to increase channel capacity and spectrum efficiency. Generally, radial uniform circular antenna array is easily to generate multimode OAM beams^6^. Recently, a number of novel methods are proposed to generate OAM carrying beams. By using traveling-wave ring-slot structure, an antenna can be used to generate four OAM modes is proposed^7^. In addition, a circularly polarized multimode patch antenna for multi-OAM-mode generation also is studied^8^.

With the development of multifunction requirements in wireless communication systems, reconfigurable antennas are required to fulfill these demands^9–11^. Polarization-reconfigurable antenna received lots of attention in modern wireless communication systems recently because of their interesting characteristics. Polarization diversity has advantages of using the frequency reuse system and can improve the performance of polarization control system^12–15^. By controlling the PIN diodes, polarization-reconfigurable antenna can be used to mitigate the polarization mismatch between the receiver and transmitter^16–18^.

In order to transmit different OAM modes by a single antenna for channel capacity improvement, we combine the concept of OAM beams multiplexing with reconfigurable antenna. In this paper, an OAM mode reconfigurable antenna is proposed, which can be used to transmit or receive mode 1 or mode −1 OAM beams by controlling the PIN diodes. There are four PIN diodes on the feeding network to control the transmitting OAM state, all PIN diodes are connected to the biasing circuit. An Advanced RISC Machines (ARM) architecture microcontroller STM32F103RCT6 is used to provide direct current (DC) power to every PIN diode individually, hence the transmitting OAM state can be controlled by the microcontroller. Moreover, we program the microcontroller and then the transmitting OAM state can be changed by a remote controller. The OAM mode reconfigurable antenna is simulated, fabricated and measured. Simulation and measurement results such as return loss, near-field and far-field radiation pattern of two operating states are given. It will be demonstrated by measurement that the proposed antenna can get a maximum 18 dB OAM diversity gain in near-field zone LOS link at 2.4 GHz. Finally, we use the proposed antenna as transmitter to encode the digital data, mode 1 OAM beam representing symbol 0 and mode −1 OAM beam representing symbol 1. At receiver side, phase gradient method is used to detect the transmitting OAM mode and then decode the data symbol. Experimental result shows that the proposed antenna is a good candidate for OAM based encoding.

## Results

### Structure of the OAM reconfigurable antenna

**Figure 1:**
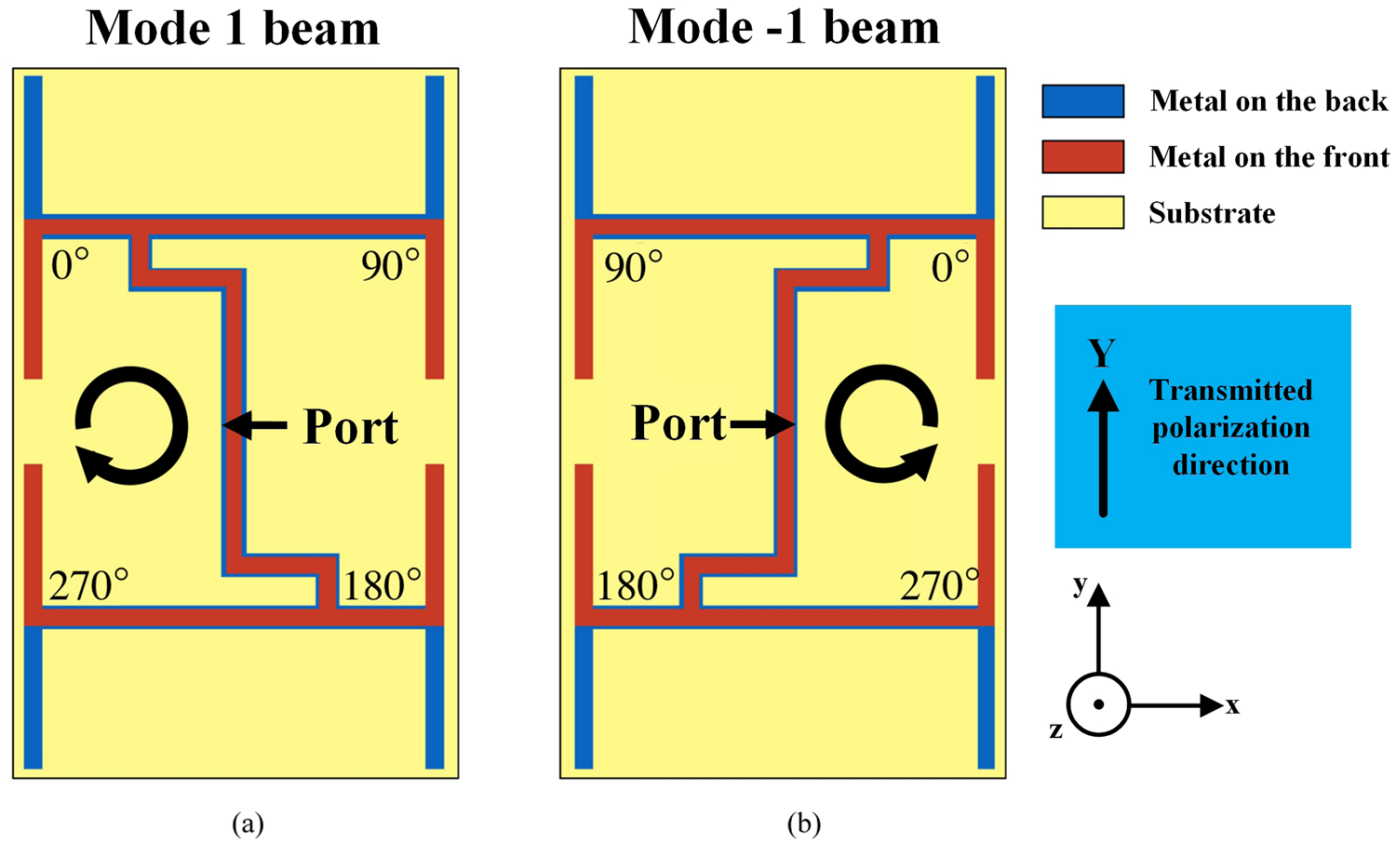
Feeding networks for OAM beam generation with different modes: (**a**) State 1 for mode 1, (**b**) State 2 for mode −1.

Uniform circular array (UCA) with successive phase shifting is a classic planar structure which can be used to generate arbitrary OAM modes^1, 6, 19^. Here we design a four-elements UCA with reconfigurable feeding network to provide two kinds of phase shifting for mode 1 and mode −1 OAM beams generation. Figure 1 shows the principle of feeding network and element placement for two operating states. The 90° phase shifting is obtained by microstrip line extension while the 180° phase shifting is achieved by simple flipping the element^20^. As a result, a 0°, 90°, 180°, 270° phase shifting for mode 1 can be obtained which is shown in Fig. 1 (a). On the other hand, the 270°, 180°, 90°, 0° phase shifting for mode −1 can be obtained by mirroring the configuration in Fig. 1 (a), which is demonstrated in Fig. 1 (b).

**Figure 2:**
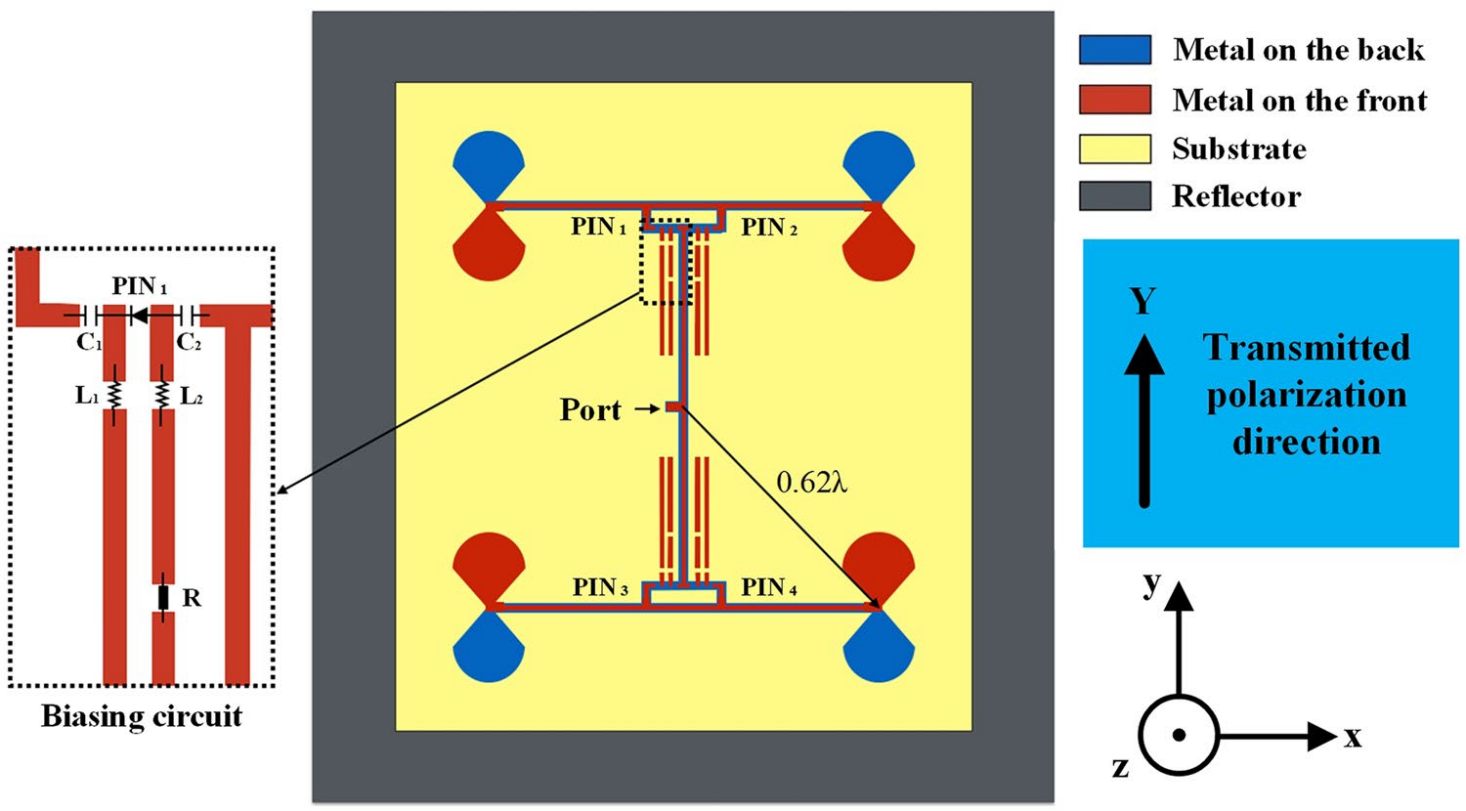
Configuration of OAM mode reconfigurable antenna and one of four biasing circuits to control operating state of PIN diode.

According to the principle illustrated in Fig. 1, the configuration of OAM mode reconfigurable antenna is illustrated in Fig. 2. The phase-shifting of the proposed configuration can be changed by controlling the operating state of 4 PIN diodes on the feeding network. Here four PIN diodes are denoted as PIN 1, 2, 3 and 4, respectively. When PIN 1 and 4 are at ON state while PIN 2 and 3 are at OFF state the proposed configuration is operating at the state shown in Fig. 1 (a) and generating mode 1 OAM beam. On the contrary, when PIN 1 and 4 are at OFF state while PIN 2 and 3 are at ON state the proposed configuration is operating at the state shown in Fig. 1 (b) and generating mode −1 OAM beam. Therefore, the proposed configuration can be used to realize the two kinds of operating state illustrated in Fig. 1, thus mode 1 or mode −1 OAM beams can be generated by the proposed configuration. The radius of the array is used to control the beam collimation, the larger the radius the more collimate of the OAM beam. Here we use 0.62 λ_2.4 GHz_ as the array radius. Four broadband radiators in the configuration, leaf-like dipoles, are used for easier matching. A metallic reflector placed λ_2.4 GHz_/4 beneath the antenna is used on one hand for gain improvement, on the other hand for avoiding the interference caused by the biasing line. Note that although the antenna has a broadband characteristic, the reconfigurable phase-shifting network is designed as narrow band. Hence the proposed configuration can only generate mode 1 or mode −1 OAM beam at 2.4 GHz.

**Figure 3:**
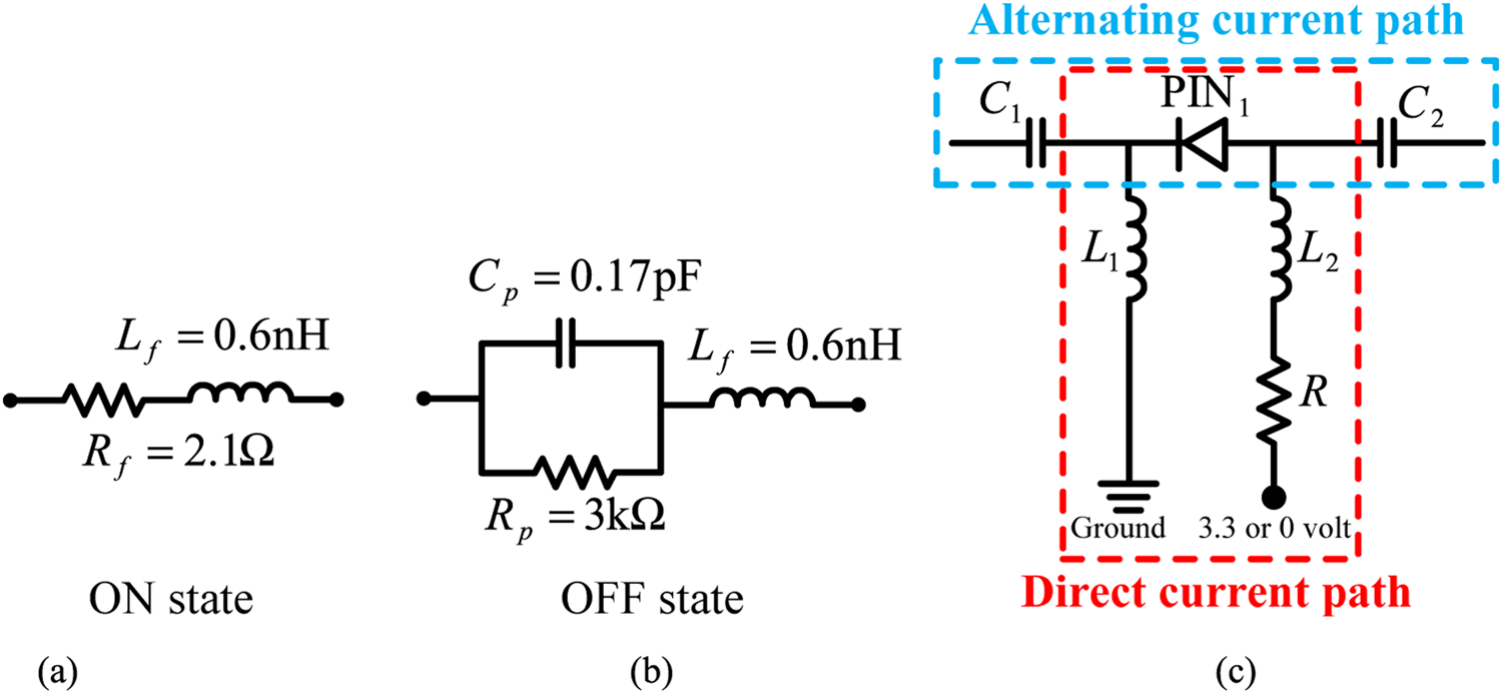
Ideal equivalent circuit models of PIN diode working at 2.4 GHz (**a**) ON state, (**b**) OFF state, (**c**) One of four biasing circuits. Blue dash line is AC path for the phase-shifting network, red dash line is DC path for DC power to control the operating state of PIN diode. When the microcontroller outputs 3.3 volt the PIN_1_ is at ON state, otherwise the PIN_1_ is at OFF state.

Here we use 4 biasing circuits to control 4 PIN diodes, one of the biasing circuits is shown in Fig. 2, and the others are the same. The main purpose of the biasing circuit is to provide direct-current power to all PIN diodes individually while do not affect the phase-shifting network. The PIN diodes we used are Bar64–02 V from Infineno Technology. Figure 3 (a) and (b) depicts the ideal equivalent circuit model of PIN diode working at ON and OFF state, respectively. These ideal equivalent circuit models are used in our simulation done by Advanced Design System (ADS). In the simulation, C_1_ and C_2_ capacitances from MuRata are 150 pF to suppress DC power, L_1_ and L_2_ inductances from MuRata are 10 nH to suppress alternating current signal, and R resistance is an adjustable resistance (0–500 ohm) to protect the DC power and can be used to optimize the return loss of the proposed antenna. Figure 3 (c) shows the alternating-current (AC) path and the direct-current path in the biasing circuit, which make sure on one hand that microwave signals would not affect DC power and on the other hand that DC power would not affect the phase-shifting network. In our prototype, a microcontroller is used to output 3.3 or 0 volt DC power for changing the operating state of four PIN diodes. Using the microcontroller, we can transmit mode 1 or mode −1 OAM beam arbitrary depend on the demand. DC power provide by the microcontroller can be changed by a remote controller which is illustrated in the antenna prototype.

### Performances of the OAM mode reconfigurable antenna

**Figure 4:**
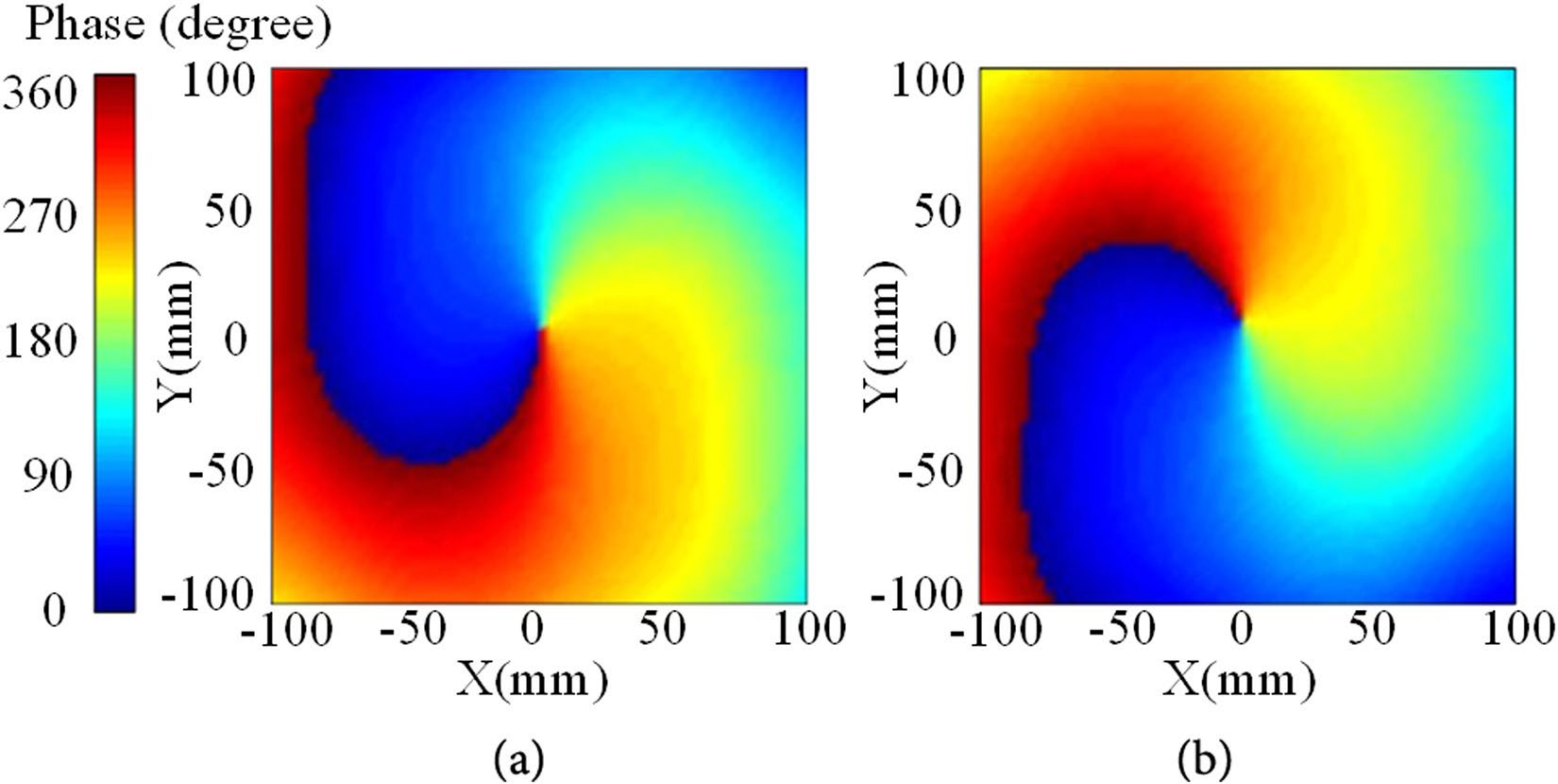
Simulation near-field Ey-phase: (**a**) State 1 for mode 1, (**b**) State 2 for mode −1.

In our simulation, the effect of PIN diode has been included. ADS is used to design the biasing circuit as illustrated above and High Frequency Structure Simulator (HFSS) is used to simulate the antenna’s radiation performances. Figure 4 shows the simulation near-field E_y_-phase at 2.4 GHz of the proposed antenna operating at state 1 for mode 1 and state 2 for mode −1.

**Figure 5:**
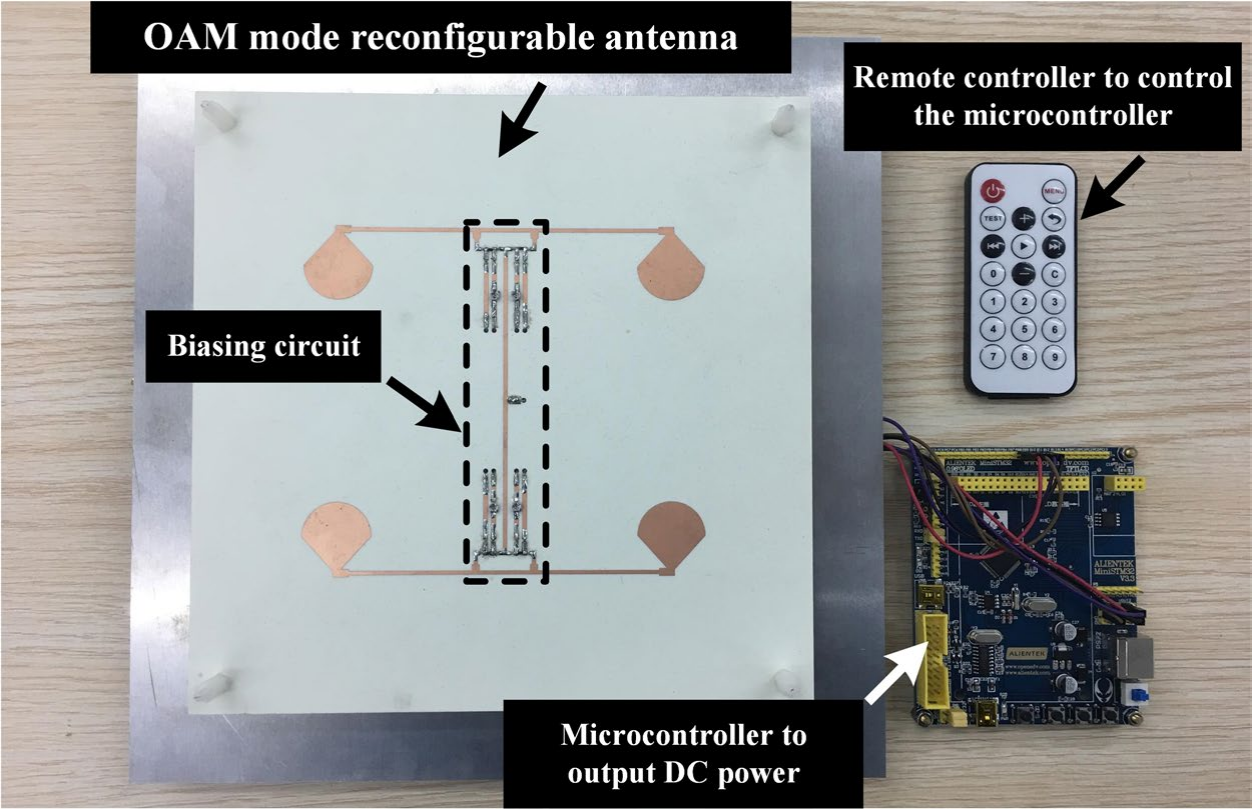
Prototype of the proposed OAM mode reconfigurable antenna.

**Figure 6:**
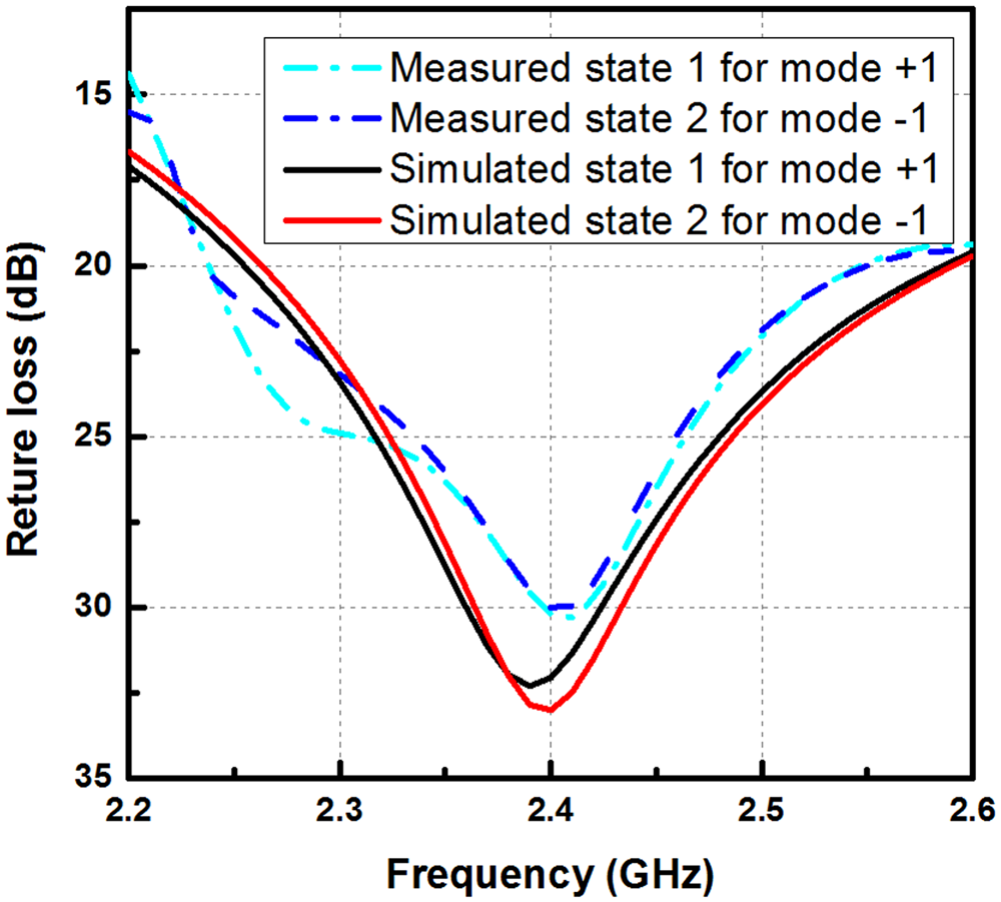
Simulation and measurement return loss of the proposed antenna at two operating states.

**Figure 7:**
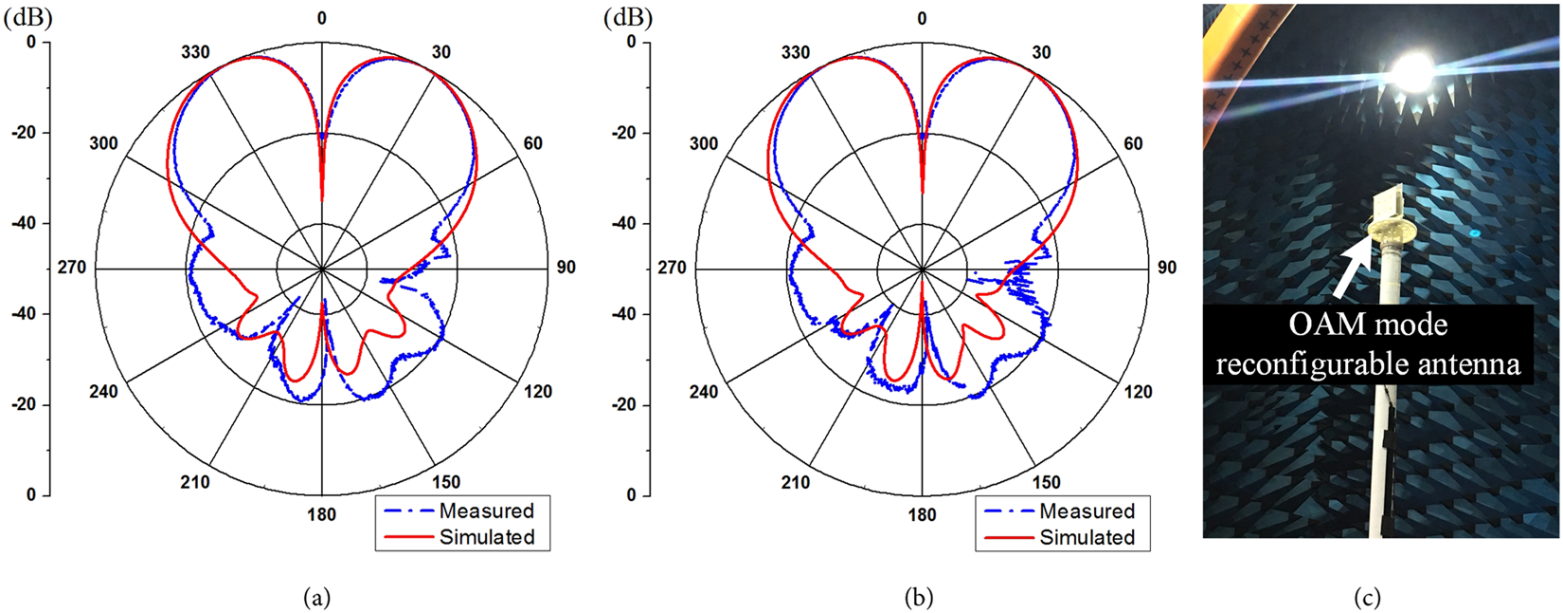
Simulation and measurement radiation pattern at E-plane of the proposed antenna at two operating states: (**a**) State 1 for mode 1, (**b**) State 2 for mode −1. (**c**) Radiation patterns measurement of the OAM reconfigurable antenna.

According to the configuration illustrated in Fig. 2, a prototype of the proposed OAM reconfigurable antenna is fabricated, shown in Fig. 5. The microcontroller is used to change the operating state of 4 PIN diodes and then change the OAM modes between mode 1 and mode −1. Figure 6 shows the simulation and measurement return loss of the proposed OAM mode reconfigurable antenna. In our simulation and fabrication, C1 = C2 = 150 pF, L1 = L2 = 10 nH and R = 500 ohm. The proposed antenna is measured by a network analyzer Agilent N5230A. The proposed antenna has a measured return loss at both states more than 15 dB from 2.2 GHz to 2.6 GHz because of the dipole element is broadband radiator. However, the phase-shifting microstrip line is designed to narrow band, therefore the proposed antenna can only generate pure OAM beam of mode 1 or mode −1 at 2.4 GHz. The maximum return loss at both state is found at 2.4 GHz. The simulation and measurement results of far-field radiation pattern of two operating states are shown in Fig. 7. Measurement of far-field radiation patterns on the fabricated antenna is carried out in a SATIMO anechoic chamber with 128 probes shown in Fig. 7 (c). Good agreement is obtained between the simulation and measurement results.

### OAM mode reconfigurable antenna for channel capacity improvement

Actually, an OAM-based MIMO radio system is equivalent to a conventional MIMO system from the view point of channel spatial multiplexing^4^. The field amplitudes experienced by the receiving elements are essentially equal but with different field phases. Using two or more antennas can capture the OAM diversity, thus improving the quality and reliability of a wireless link. Different OAM modes provide different phase distributions in the receiving zone. If and/or when signal degrades, by switching the transmitting OAM modes, the receiving signal may get an improvement because of different receiving phases. The more different transmitting OAM modes can be switched (including mode 0, i.e., the normal beam), the better chance can be got to improve the degraded receiving signal without any signal post-processing.

**Figure 8:**
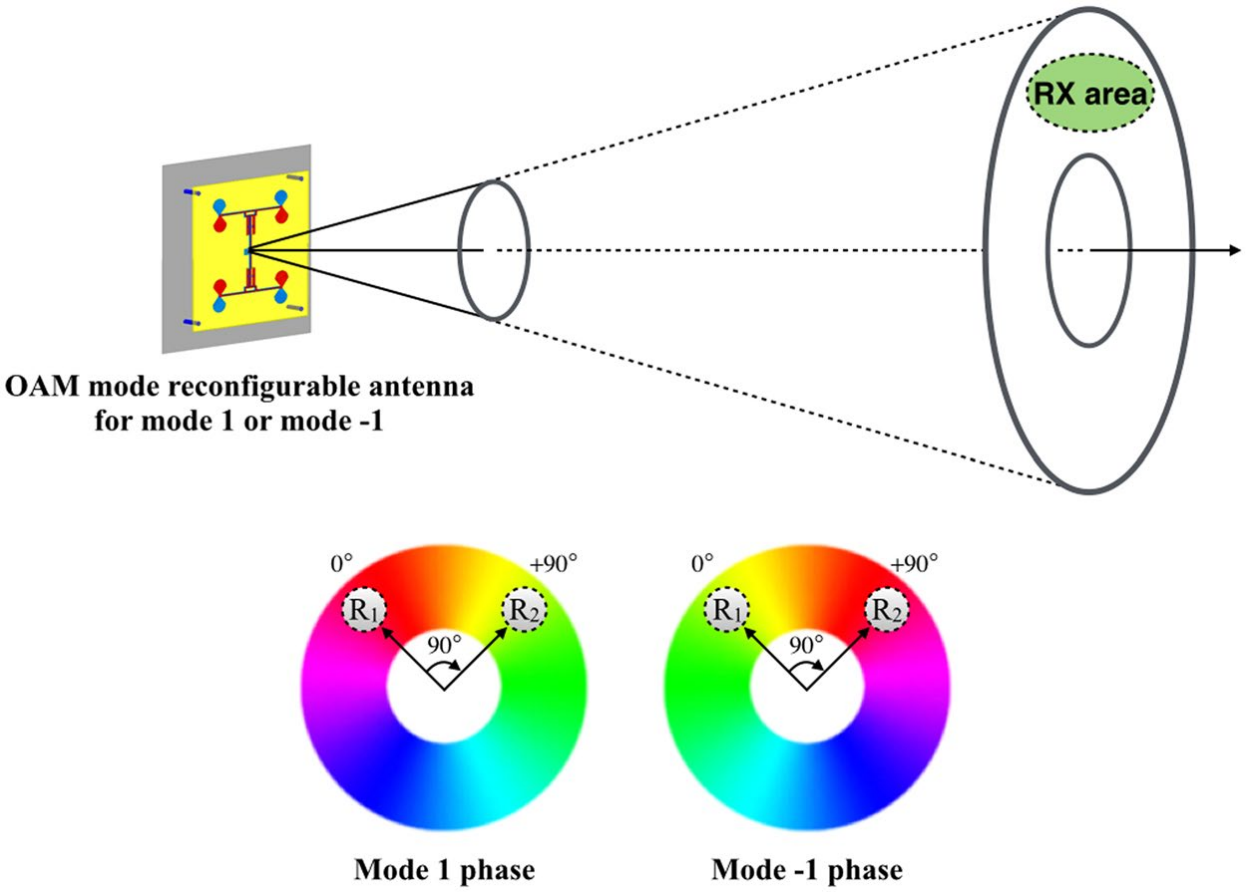
Illustration of an OAM mode reconfigurable antenna to improve the received signal quality. A two-element array fed with different phases is used to capture the OAM diversity.

Here is an ideal example to illustrate how the proposed OAM mode reconfigurable antenna improves the received signal quality. Suppose the receiver (RX) is a planar array of two identical elements denoted as R_1_ and R_2_ fed with phases 0° and +90°, respectively. They must be placed in a doughnut-shaped zone but may be off-axis placed as illustrated in Fig. 8.

In this case, the two elements have the same receiving amplitudes but with different receiving phases. At the receiver, the signal is degraded when the OAM mode is mismatched, while the signal is improved when the OAM mode is matched. When the transmitter is transmitting the beam with mode 1, two paths introduce additional +90° phase shift before being received; therefore, the receiving signal is degraded. On the other hand, when transmitting antenna is transmitting the beam with mode −1, two paths introduce additional −90° phase shift before being received, thus the receiving signal is improved. As a result, we can switch the transmitting OAM modes to improve the quality and reliability of a wireless link. Actually, the OAM diversity can be considered as a spatial diversity without any signal processing.

**Figure 9:**
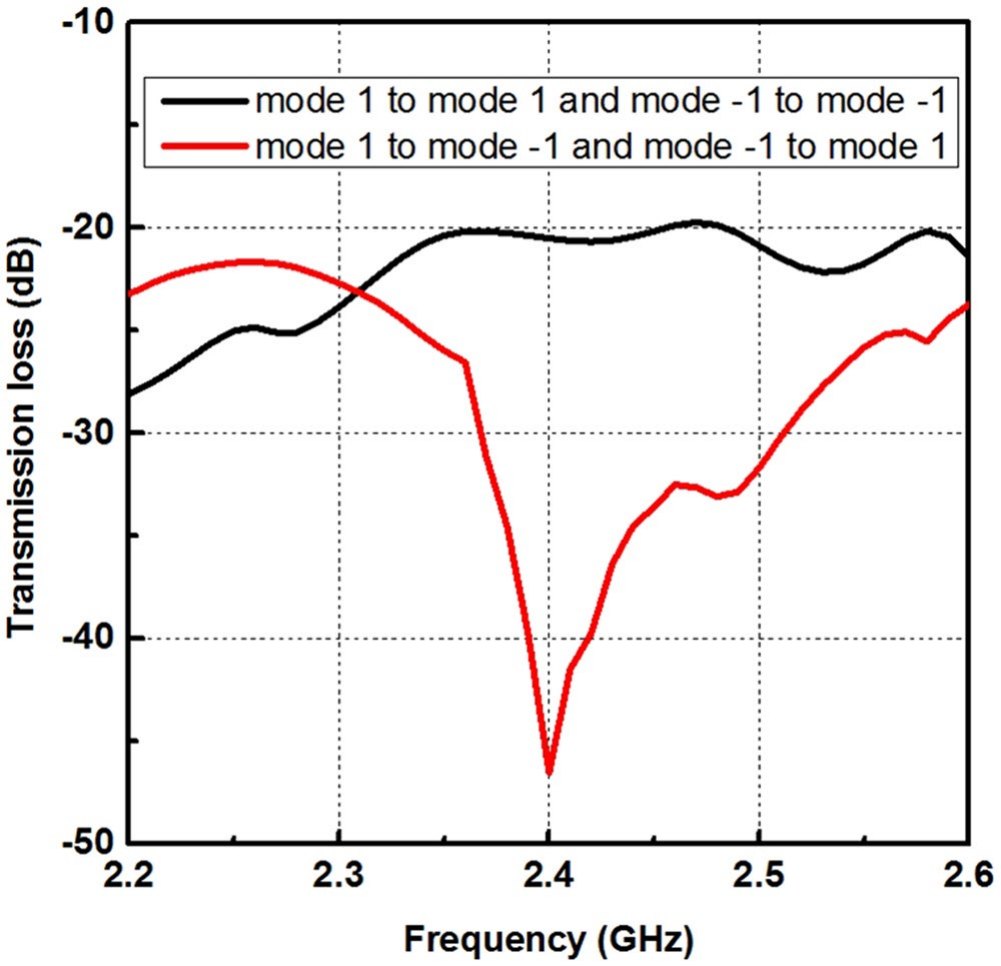
Simulated transmission loss with two proposed antennas as transmitter and receiver, respectively.

For simplicity, here we use the OAM mode reconfigurable antenna as a receiver to capture the OAM diversity. We build a near-field line-of-sight (LOS) link, in which two proposed antennas are used as transmitter and receiver respectively. They are perfectly aligned to each other and separated by 2.5 meters away. In order to assess the OAM diversity gain of the proposed link, the transmission loss between the transmitter and the receiver in the mode-matched state and the mode-mismatched state are simulated by HFSS. The simulation results are shown in Fig. 9.

**Figure 10:**
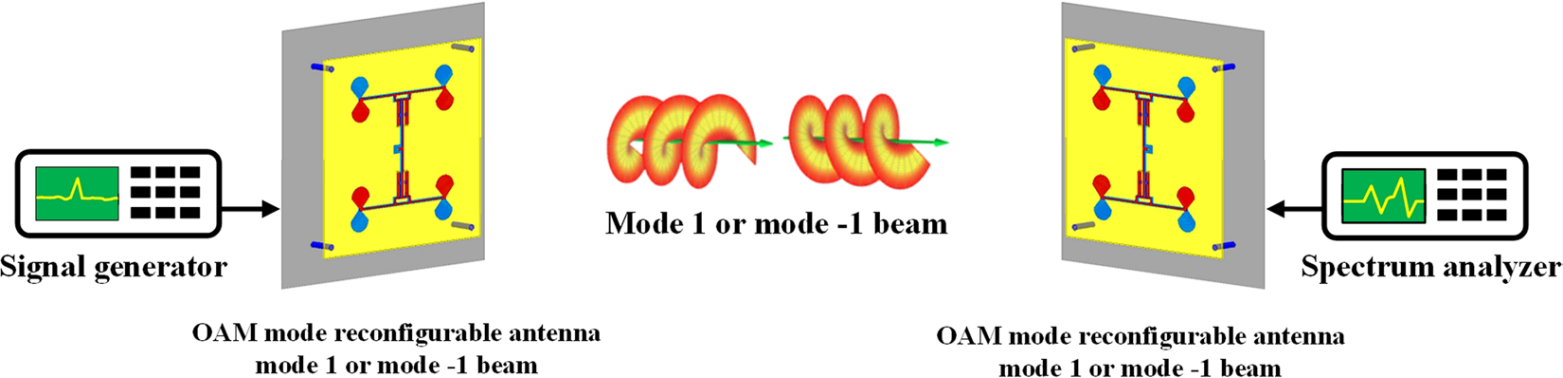
Configuration of the proposed link for OAM diversity gain measurement.

Moreover, a prototype of the proposed antenna connected to the signal generator Anritsu MS2665C (generate 2.4 GHz wave with power 0 dBm) works as a transmitter, and another prototype of the proposed antenna connected to the spectrum analyzer Anritsu MG3692B works as a receiver, they are aligned just like in our simulation of transmission loss^21^, and then we change the operating state of the transmitter and receiver by the microcontroller, shown in Fig. 10. The measurement results are shown in Table I.

**Table 1:** Received average power by measurement of the proposed link at 2.4 GHz.

Case (at 2.4 GHz)	Received average power
mode 1 to mode 1 (Matched)	−15 dBm
mode −1 to mode −1 (Matched)	−14 dBm
mode 1 to mode −1 (Mismatched)	−33 dBm
mode −1 to mode 1 (Mismatched)	−35 dBm

From the simulation results we can see that when the transmitter and receiver are operating at the same state we can get a higher power about 25 dB than when they are working at different states. This means that in the ideal case we can get a 25 dB OAM diversity gain at 2.4 GHz by the proposed antenna. In the measurement, the mismatched received signal power is about −33 dBm and the matched received signal power is about −15 dBm. Hence, we can get 18 dB OAM diversity gain by switching the transmitting OAM modes between mode +1 and mode −1 in the measurement. The differences between measurement and simulation results are due to the misalignment of the link^22^. It should be noted that using two or more antennas as receiver can also get the OAM diversity gain, as shown in Fig. 8.

### OAM mode reconfigurable antenna for digital data encoding

**Figure 11:**
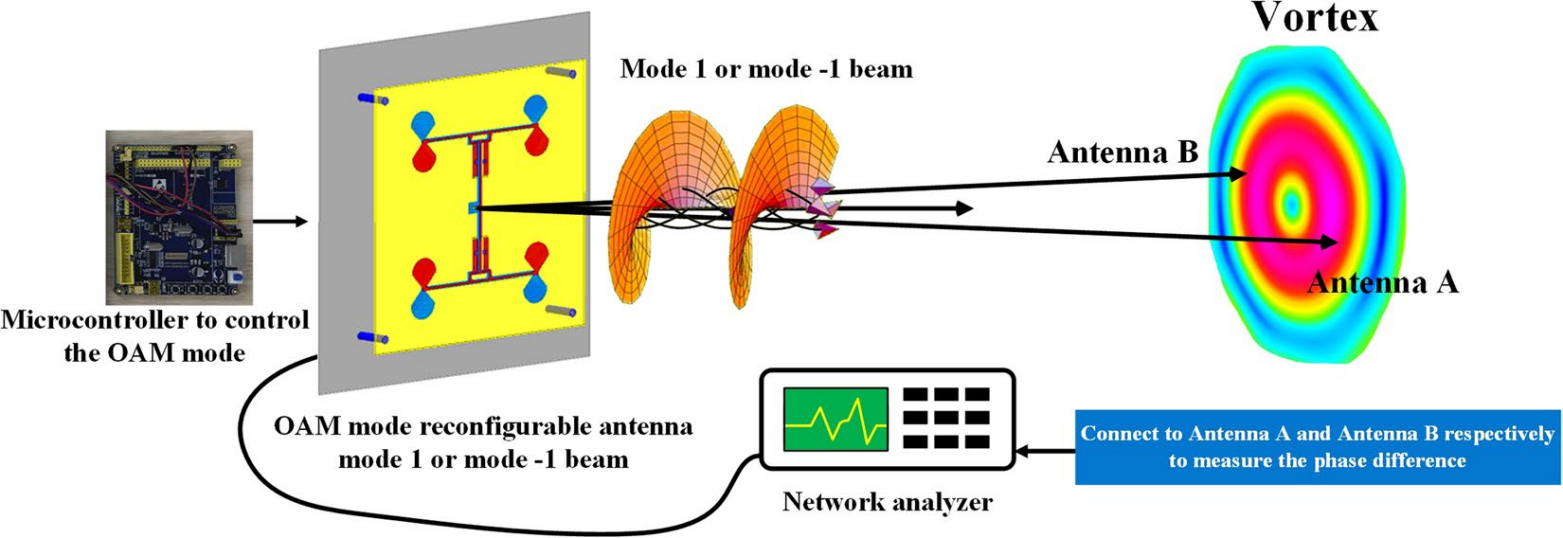
Phase gradient method to detect the OAM mode transmitted by the proposed antenna. Antenna A and B are connected to the network analyzer respectively to measure the phase difference *ϕ*.

By using phase gradient method we can separate the two OAM modes from their phase difference^23^. Comparing the signal phases from two antenna, antenna A and B, as described in Fig. 11. EM waves propagating along the two paths from the source to the two receiving antennas A and B, 2.5 meters as well, acquired a relative phase difference between the two receiving antennas *ϕ* due to the beam vorticity. In theory *ϕ* = +*π* when the proposed antenna is transmitting mode 1 beam and *ϕ* = −*π* when the proposed antenna is transmitting mode −1 beam.

**Figure 12:**
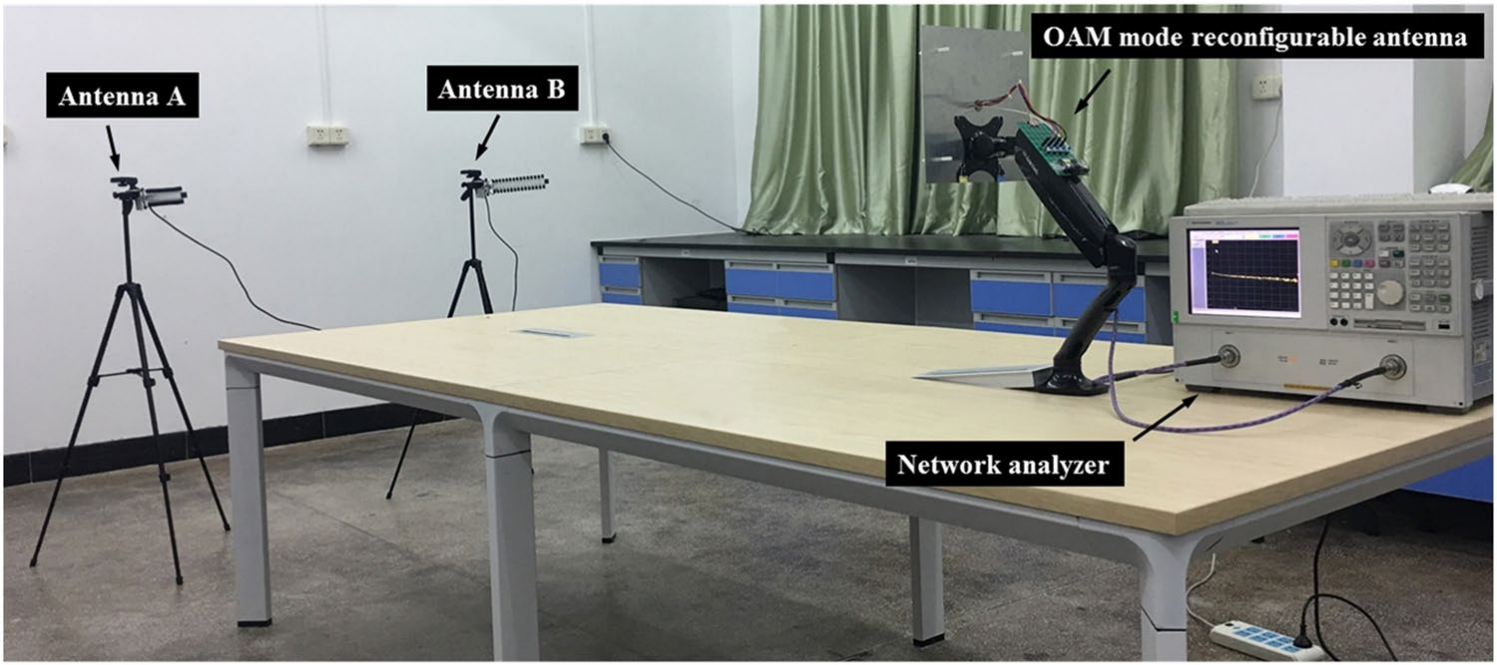
Experiment of the phase gradient method to detect OAM mode.

In our experiment, we use a pair of commercial Yagi-Uda antennas working at 2.4 GHz which are connected to a network analyzer to measure the phase gradient, and we change the OAM mode by the microcontroller, shown in Fig. 12. The measurement results are shown in Table 2.

**Table 2:** Measurement relative phase difference *ϕ* for mode 1 and mode −1, which are mapped to symbol 0 and 1, respectively.

Case (at 2.4 GHz)	Simulation phase difference ϕ	Measurement phase difference ϕ
mode 1 (symbol 0)	+180°	+157°
mode −1(symbol 1)	−179°	−163°

The disagreement between the measurement results and theory results are due to the misalignment of the link and the reflection waves from the ground. However, we can still distinguish mode 1 from mode −1 by the tolerance results. From the measurement results we find that the proposed system can be used to detect mode 1 or mode −1. Therefore, data symbols 0 and 1 can be encoded by the proposed OAM reconfigurable antenna^5^, shown in Table 2. For example, data stream is consist of symbol 0 and symbol 1, which are represented by mode 1 and mode −1 OAM beams, respectively. Every time slot is used to transmit a mode 1 or mode −1 OAM beam representing symbol 0 or symbol 1, the microcontroller is used to control the transmitting OAM mode according to the data stream. On the other side, phase gradient method is used to detect the data stream, and relative phase difference is calculated to decode the data stream. The proposed OAM mode reconfigurable antenna is a good candidate for wireless data encoding.

## Discussion

Combining the concept of OAM mode multiplexing for channel capacity improvement with reconfigurable antenna, an OAM mode reconfigurable antenna is firstly proposed in this paper. A microcontroller is used to control the operating state of PIN diodes, and then control the transmitting OAM mode. Moreover, we program the microcontroller that we can change the transmitting OAM mode between mode 1 and mode −1 arbitrary by the remote controller. The antenna’s performance such as near-field phase, far-field radiation pattern and return loss are given, which show that the proposed OAM mode reconfigurable antenna can indeed generate OAM mode 1 or mode −1 by a single port.

On one hand, a polarization-reconfigurable antenna can increase the channel capacity by changing the polarization states. Similarly, an OAM mode reconfigurable antenna can improve the channel capacity by the switchable OAM modes between the receiver and transmitter. The proposed antenna can be used to provide the OAM diversity with only a single port, which is different from the existing multi-OAM-mode antennas with multi ports. The existing multi-OAM-mode antennas increase the channel capacity by multiplexing different data streams at different OAM mode beams. However, our proposed OAM reconfigurable antenna increase the channel capacity by switching the transmitting OAM mode and improve the signal quality. In our experiment, when signal is degraded because of OAM mode mismatch, we can switch the transmitting OAM mode and get an 18 dB OAM diversity gain. The more OAM modes can be switched by the OAM mode reconfigurable antenna, the better chance we can get to improve the signal quality. Hence, the OAM mode reconfigurable antenna is a good candidate to increase the channel capacity in near-field zone LOS link.

On the other hand, OAM-mode based encoding was firstly proposed in 2014. Due to the OAM mode switchable property of the proposed antenna, it can be used as a transmitter to encode the digital data by different OAM modes. Every time slot a data symbol corresponding to a unique OAM mode that is transmitted by the proposed antenna, which can be easily achieved by the microcontroller through changing the OAM mode. The receiver we use in our experiment is two commercial Yagi-Uda antennas to decode the digital data by phase gradient method. Despite of the error between the theory results and experimental results, we can still distinguish OAM mode 1 from mode −1 in our experiment. Therefore, the OAM mode reconfigurable antenna is a good candidate for OAM based encoding.

## Method

The return loss, near-field phase, radiation patterns of the OAM mode reconfigurable antenna, and the transmission loss between the transmitter and receiver are simulated by High Frequency Structure Simulator (HFSS 14.0) and Advanced Design System (ADS 2009). The prototype is printed on a Rogers 4350B substrate. The adjustable resistance is measured by a digital multimeter Victor VC890D. The return loss of the prototype is measured by a network analyzer Agilent N5230A. The far-field radiation patterns are measured in a SATIMO anechoic chamber with 128 probes. In the inter-mode crosstalk experiment, the transmitter is connected to a signal generator Anritsu MS2665C, and the receiver is connected to a spectrum analyzer Anritsu MG3692B, the transmitter and receiver are separated 2.5 meters. In the OAM mode encode experiment, one port of a network analyzer Agilent N5230A is connected to the proposed OAM mode reconfigurable antenna, an Advanced RISC Machines (ARM) architecture microcontroller STM32F103RCT6 is used to change OAM mode between mode 1 and mode −1 transmitting by the proposed antenna. Another port of the network analyzer is connected to two identical commercial Yagi-Uda antenna one by one to measure the relative phase difference. The operating frequency of commercial Yagi-Uda antenna is at 2.4 GHz and with a gain 16 dBi.
